# Dataset on applying HPMC polymer to improve encapsulation efficiency and stability of the fish oil: In vitro evaluation

**DOI:** 10.1016/j.dib.2020.106111

**Published:** 2020-08-04

**Authors:** I.S.M. Zaidul, T.K. Fahim, F. Sahena, A.K. Azad, M.A. Rashid, M.S. Hossain

**Affiliations:** a Faculty of Pharmacy, International Islamic University Malaysia, Kuantan Campus, 25200 Kuantan, Pahang, Malaysia; b Faculty of Science, International Islamic University Malaysia, Kuantan Campus, 25200 Kuantan, Pahang, Malaysia; c Division of Environmental Technology, School of Industrial Technology, Universiti Sains Malaysia, Malaysia

**Keywords:** Fish oil, Encapsulation, Particle formation, Spray drying, Hydroxypropyl methylcellulose, Peroxide value, Simulated fluid

## Abstract

Data examines the effect of hydroxypropyl methylcellulose (HPMC) HPMC15 cP, and HPMC 5 cP polymer composition on the physicochemical traits of encapsulated oil made using lab scale spray drying (180 °C). The data found showed that the properties of the reconstituted fish oil powder are significantly affected by the polymer's composition and ratio (*p* < 0.05). In this experiment, powder with the particle sizes below 60 μm was produced and it was observed that HPMC is a good emulsifier for all formulations and the encapsulation efficiency is high with 75.21% for AF1 formulation. It was also observed that the process of fish oil encapsulation employed by HPMC 5 cP produce a more volatile oil powder, while encapsulation with HPMC 15 cP produced a more stable fish oil powder. These finding shows that the utilisation of HPMC as a polymer to encapsulate fish oil can produce a more efficient and stable compound.

Specifications table

**Table:** 

Subject area	Pharmaceutical Science
Specific subject area	Drug discovery, Pharmaceutical Technology, Industrial and Manufacturing Engineering
Type of data	Table, image, graph, figure
How data were acquired	Homogeniser (Ultra-Turrax® T25), lab scale spray dryer (Lab Plant SD06A, UK), viscometer (DV-III Ultra, Brookfield, USA), laser diffraction using laser particle size analyser BT-9300H (Dandong Bettersize Instruments, Dandong, China), moisture analyser (A&D MS-70, Japan), laser diffraction particle size analyser (Malvern 2000 mastersizer, Malvern Instruments Co., Worcestershire, UK), field emission scanning electron microscope (JEOL JSM-7800F, Japan), A/S Niro Atomiser, rotary evaporator.
Data format	Raw, Analysed and graph
Parameters for data collection	Preparation of fish oil emulsion with different ratios of HPMC 15 cP and 5 cP polymer using employed spray drying technique for microencapsulation.
Description of data collection	First, fish oil was converted into emulsion by mixing polymers in a specific condition and characterised by reviewing it based on various criteria, such as viscosity and droplet size. It was then converted into powder by spray drying. The powder was collected from a Schott bottle which was attached at the bottom of the cyclone separator and stored in an amber glass bottle at 4 °C. It was then evaluated for its moisture content, encapsulation efficiency, particle size distribution, density, flowability, cohesiveness, particle density, porosity and Peroxide value. The digestion ability was simulated in simulated gastric fluid and simulated intestinal fluid in vitro. Data were collected very precisely, before being analysed and reported.
Data source location	Faculty of Pharmacy, International Islamic University Malaysia, 25200 Kuantan, Pahang, Malaysia and University Putra Malaysia, Serdang, Malaysia.
Data accessibility	All the data are provided in this manuscript
Related research article	Microencapsulation of Fish Oil Using Hydroxypropyl Methylcellulose as a Carrier Material by Spray Drying, Journal of Food Processing and Preservation, https://doi.org/10.1111/jfpp.12591

Value of data

• This data's finding could benefit various parties as it demonstrates that the use of spray drying to encapsulate fish oil with HPMC 5 cP and HPMC 15 cP could produce more stable and efficient microencapsulated fish oil.
• In this regard, it was observed that converting liquid fish oil into powder and encapsulating the oil using the spray drying technology produce higher efficiency and stability without altering its physiochemical characteristics.
• This will benefit the food and health industry, specifically in the manufacturing of food, nutraceuticals and pharmaceuticals products where this process can be used to produce Omega-3 fish oil in powder form as an alternative for soft-gel encapsulated fish oil.
• Powdered fish oil can also be used to produce more health products such as Baby Food. Moreover, this data's findings have shown that HPMC and spray drying have good binding capacity and easily available.
• This could guide researchers on the use of spray drying and HPMC to formulate and encapsulate other oil for nutraceutical and pharmaceutical purposes. At the same time, the research data could provide further insights into the process of encapsulating fish oil and how it could be used in large-scale production and improve current practices.

## 1 Data description

Table 2 present oil droplets’ mean diameter and the viscosity of the emulsions. It is notable that the emulsions have different solid concentrations. Based on the data, the mean diameters of the oil droplets in all formulations are significant at p value (*p*< 0.05). Meanwhile, the droplets range from 3.72 to 19.35 µm in size, while the fish oil powder has between 3.39 and 7.28% of moisture content (Table 3). Subsequently, the surface oil content and total oil content were considered in calculating the efficiency of the encapsulation. The data presented in Table 3 show that the oil encapsulation efficiency of powder is between 62.13 and 75.21%.

**Fig. 1:**
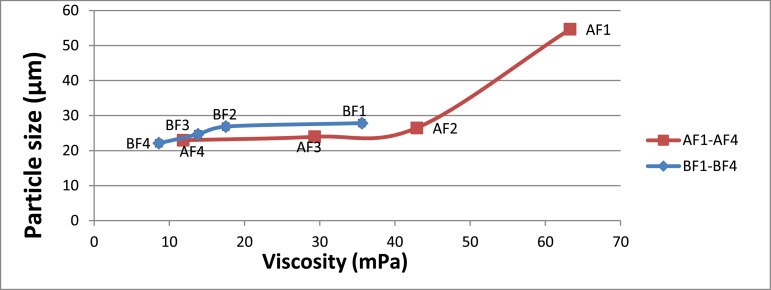
Effect of solution viscosity on particle size.

**Fig. 2:**
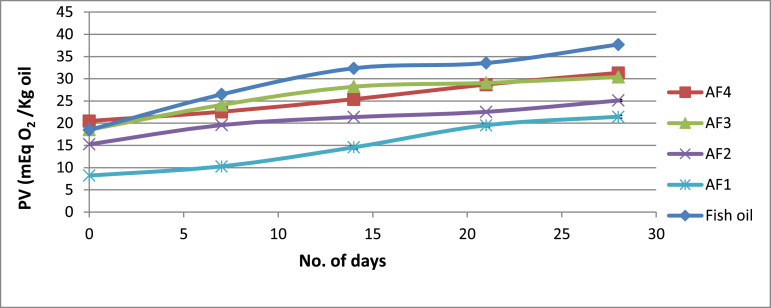
Effect of storage time on the peroxide value of formulation AF1, AF2, AF3 & AF4.

**Fig. 3:**
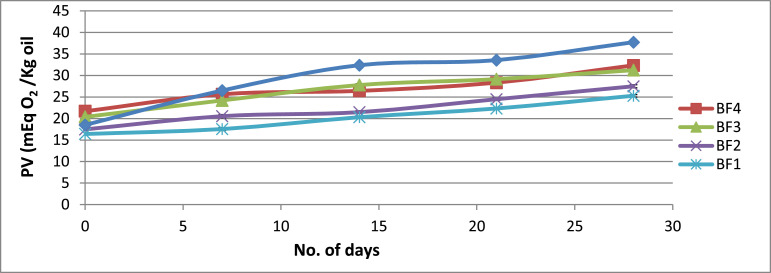
Effect of storage time on the peroxide value of formulation BF1, BF2, BF3 & BF4.

Another notable observation is that the HPMC type and composition significantly influence the fish oil powder's encapsulation efficiency (*p* < 0.05) with the encapsulated oil particles range between 22.07 µm and 54.67 µm. Fig. 1 illustrates how powder's particle sizes are affected trough the viscosity of the HPMC 5 cP and HPMC 15 cP viscosity. Meanwhile, Table 4 shows that of the particles’ tapped and bulk densities are influenced by composition and the wall material ratio. Another significant finding is that the Carr index values are between 7.69% to 21.87% and this data is significantly linked to the powder's particle size (*p* < 0.05). Table 4 illustrates that The Housner ratio ranges between 1.08 and 1.28 while Table 5 shows that 18.27–67.40% of oil were released in SGF. It could also be observed the PV of AF4 increased to 31.08 mEq O2/Kg oil from 20.35 mEq O2/Kg oil in the duration of 28 days (4 weeks). At the same time, the formulation of AF3 improved to 30.65 mEq O2/Kg oil and similarly, the formulation of AF2 increased to 25.28 mEq O2/Kg oil (Fig. 2). Meanwhile, as shown in Fig. 3, the use of advanced inlet drying air temperature raised PV in BF3 (32.74 mEq O2/Kg oil) and BF4 (31.61 mEq O2/Kg oil) while Fig. 5 shows that in comparison to fish oil encapsulated using HPMC 5 cP, the fish oil encapsulated with HPMC 15 cP showed less deformation as well as less wrinkled and dented surface.

## 2 Experimental design, materials, and methods

### 2.1 Materials

Incepta Pharmaceuticals Ltd, Bangladesh kindly donated the HPMC (Methocel E5 Premium LV and Methocel E15 Premium LV) used in this data. At 20 °C, the HPMC recorded the viscosity of E15 and E5 (2% w/v solution). Meanwhile, to adhere to the US requirements, the materials were sourced from Sigma–Aldrich Inc, St Louis, Missouri; 20–30% Omega-3 Fish oil was used for this data along with pancreatic collected from pepsin derived from porcine mucosa and porcine pancreas.

### 2.2 Preparation of microencapsulated fish oil

Table 1 presents A and B sequences formulations set for this data. Both formulation series were prepared using plasticiser [Polyethylene glycol (PEG) 6000] along with the HPMC 5 cP and HPMC 15 cP polymers. Moreover, Ultra-Turrax® T25 basic (IKA Labortechnik, Germany) was used to homogenize at 200 g of the formulations at 14,000 rpm for 12 min

**Table 1:** Formulations for preparing microencapsulated fish oil.

*Series*	*Formulations*	*HPMC 5* *cps (g)*	*HPMC 15* *cps (g)*	*PEG 6000 (g)*	*Fish oil (g)*	*Distilled Water (g)*	*Solid content (wt.%) Ref.*[1]*[25]*
1	AF1	–	10	0.5	10	179.5	10.25
	AF2	–	7.5	0.5	10	182	9.0
	AF3	–	5	0.5	10	184.5	7.75
	AF4	–	2.5	0.5	10	187	6.5
2	BF1	10	–	0.5	10	179.5	10.25
	BF2	7.5	–	0.5	10	182	9.0
	BF3	5	–	0.5	10	184.5	7.75
	BF4	2.5	–	0.5	10	187	6.5

After the homogenisation, process was completed, a Lab Plant SD06A lab scale spray dryer from the UK, was used to spray dry the formulations. The spray dryer was fitted with an auto jet de-blocking system with a 215 mm OD x 500 mm ID compressor, spray atomiser compressor, and a 0.5 mm atomiser. The pump pressure was fixed at 407.1 mL/hr while the air velocity was fixed at 4.1 m/s during the process to allow the emulsion to be transported into the expansion vessel from the feed. Furthermore, the outlet temperature was fixed at 80 ± 1 °C while the inlet at180 °*C* ± 1 °C. Then encapsulated oils were obtained via Schott bottle which was attached to the bottom of the cyclone separator. They were then stored at 4 °C.

### 2.3 Characterisation of fish oil emulsion

#### 2.3.1 Viscosity of the emulsion

As shown in Fig. 1 and Table 2, a viscometer sourced from DV-III Ultra, Brookfield, was used in measuring the viscosities of the emulsion. The viscometer is fitted with spindle SC4–18.

**Table 2:** Viscosity and droplet size of emulsions with different solid content.

*Formulations*	*Total solid content (wt.%)*	*Viscosity (mPa. s)*	*Droplet size, D_4,_*_3_*(µm)*
AF1	10.25	63.27 ± 0.15 ^a^	3.72 ± 0.03 ^a^
AF2	9.0	42.90 ± 0.10 ^b^	7.20 ± 0.02 ^b^
AF3	7.75	29.30 ± 0.10 ^c^	12.77 ± 0.02 ^c^
AF4	6.5	11.83 ± 0.06 ^d^	13.66 ± 0.02 ^d^
BF1	10.25	35.60 ± 0.10 ^a^	6.76 ± 0.03 ^a^
BF2	9.0	17.50 ± 0.10 ^b^	11.88 ± 0.01 ^b^
BF3	7.75	13.80 ± 0.10 ^c^	17.83 ± 0.04 ^c^
BF4	6.5	8.60 ± 0.10 ^d^	19.35 ± 0.04 ^d^

Values are average of triplicate (*n* = 3) analyses ± standard deviation.

^a,b,c,d^ Letter within each column is significantly different at *p* < 0.05 using Tukey's HSD post-hoc test.

#### 2.3.2 Emulsion droplet size

Laser diffraction measured the emulsion droplets’ size. The process involved laser particle size analyser BT-9300H produced by Dandong Instruments, Dandong, China. Table 2 presents the volume weighted mean, D, which represents the emulsion droplet size.

### 2.4 Characterisation of fish oil powder

#### 2.4.1 Moisture content

As shown in Table 3, similar to Karim et al., [1] a moisture analyser (A&D MS-70, Japan) was used to determine the moisture content of the powder produced via the spray dried approach.

**Table 3:** Characteristics of encapsulated powder of different formulations.

*Formulation*	*Moisture (wt.%)*	*Encapsulation Efficiency (%)*	*Particle size, D_4,_*_3_*(µm)*	*Wettability (*min*)*
AF1	3.85 ± 0.04 ^a^	75.21 ± 0.75 ^a^	54.67 ± 0.09 ^a^	20.67 ± 0.58 ^a^
AF2	5.07 ± 0.05 ^b^	71.69 ± 0.69 ^b^	26.49 ± 0.06 ^b^	18.67 ± 0.58 ^b^
AF3	3.39 ± 0.03 ^c^	69.72 ± 0.77 ^b^	23.96 ± 0.04 ^c^	14.67 ± 0.58 ^c^
AF4	4.89 ± 0.04 ^d^	66.31 ± 0.88 ^c^	22.97 ± 0.02 ^d^	13.33 ± 0.58 ^c^
BF1	4.35 ± 0.04 ^a^	67.44 ± 0.98 ^a^	27.84 ± 0.04 ^a^	15.67 ± 0.58 ^a^
BF2	5.83 ± 0.05 ^b^	65.81 ± 0.08 ^a^	26.87 ± 0.04 ^b^	11.67 ± 0.58 ^b^
BF3	7.28 ± 0.04 ^c^	63.32 ± 0.78 ^b^	24.66 ± 0.05 ^c^	9.33 ± 0.58 ^c^
BF4	4.83 ± 0.05 ^d^	62.13 ± 0.54 ^b^	22.07 ± 0.04 ^d^	7.83 ± 0.29 ^c^

Values are average of triplicate (*n* = 3) analyses ± standard deviation.

^a,b,c,d^ Letter within each column is significantly different at *p* < 0.05 using Tukey's HSD post-hoc test.

#### 2.4.2 Determination of microencapsulation efficiency

The procedures highlighted in studies like Karim et al., [1] were slightly modified to determine the total oil content.

#### 2.4.3 Particle size distribution

Laser diffraction measured the particle size distribution of the sample. The process used a Malvern 2000 master siser particle size analyser which was produced by Malvern Instruments Co., Worcestershire, UK. In reference to Scirocco (2000), the analyser was equipped with an automated dry powder dispersion unit. Table 3 presents the volume weighted mean, D4,3, which show the particle size distribution of the sample.

#### 2.4.4 Particle surface morphology

**Fig. 4:**
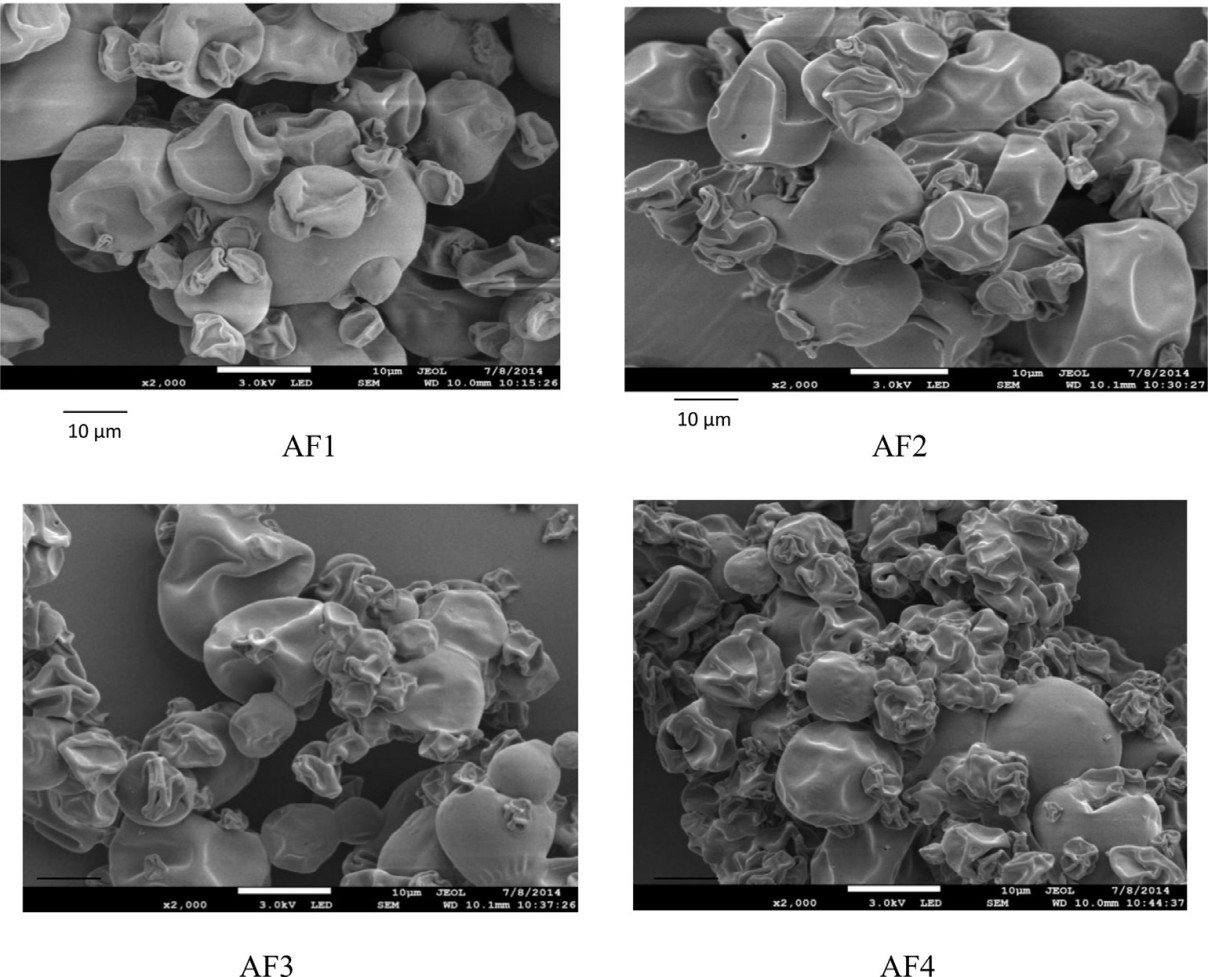
Morphology of encapsulated powder with HPMC 15 cps at different concentration.

**Fig. 5:**
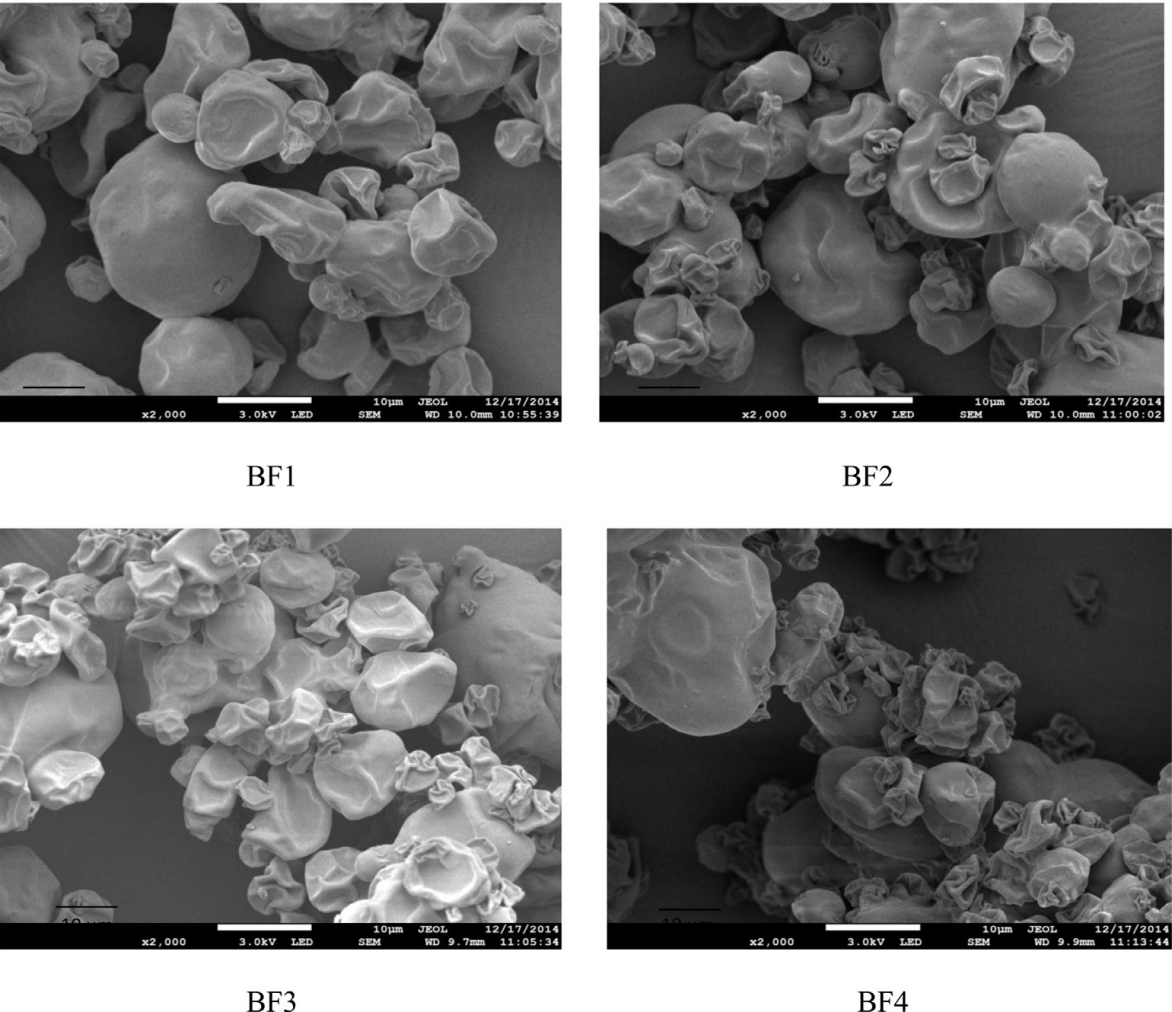
Morphology of encapsulated powder with HPMC 5 cps at different concentration.

FESEM (JEOL JSM-7800F, Japan) was utilised to analyse the morphologies of the particles. As illustrated in Figs 4–5, during the process, double-sided adhesive carbon tapes were used to mount the dried powder on the specimen stubs before they were platinum coated and examined at 1–3 kV with the magnification between 500x and 10,000x.

#### 2.4.5 Wettability of powder

The method proposed in Fuchs et al., [6] was slightly modified and used to determine the wettability of the fish oil power. Then, as shown in Table 3, 1 g of the sample were scattered over the surface of 100 mL distilled water without agitation. The temperature was set at 20 °C.

#### 2.4.6 Bulk density and tapped density of powder

The experiment presented in Jinpong et al., [5] was slightly modified to sample's the bulk density (Table 4).

**Table 4:** Characteristics of encapsulated powder of different formulations.

*Formulation*	*Bulk Density (g mL^−1^)*	*Tapped Density (g mL^−1^)*	*Flowability & Cohesiveness*	
			*Carr index (%)*	*Housner ratio*
AF1	0.133 ± 0.001 ^a^	0.149 ± 0.003 ^a^	10.90 ± 1.23 ^a^	1.12 ± 0.01 ^a^
AF2	0.121 ± 0.001 ^b^	0.143 ± 0.001 ^b^	15.60 ± 0.26 ^a^	1.18 ± 0.01 ^a^
AF3	0.118 ± 0.001 ^b^	0.137 ± 0.002 ^b^	14.07 ± 1.88 ^a^	1.16 ± 0.03 ^a^
AF4	0.115 ± 0.001 ^b^	0.130 ± 0.003 ^b^	11.03 ± 2.13 ^a^	1.12 ± 0.03 ^b^
BF1	0.173 ± 0.003 ^a^	0.187 ± 0.003 ^a^	7.83 ± 0.35 ^a^	1.08 ± 0.01 ^a^
BF2	0.145 ± 0.008 ^b^	0.157 ± 0.003 ^b^	7.69 ± 3.94 ^a^	1.08 ± 0.05 ^a^
BF3	0.129 ± 0.002 ^c^	0.147 ± 0.003 ^c^	11.98 ± 2.88 ^a^	1.14 ± 0.04 ^a^
BF4	0.090 ± 0.004 ^d^	0.116 ± 0.002 ^d^	21.87 ± 4.09 ^b^	1.28 ± 0.09 ^b^

Values are average of triplicate (*n* = 3) analyses ± standard deviation.

^a,b,c,d^ Letter within each column is significantly different at *p* < 0.05 using Tukey's HSD post-hoc test.

#### 2.4.7 Flowability and cohesiveness of powder

The procedures presented in Karim et al., [1], shown in Table 4, was adopted the determine the sample's flowability and cohesiveness (Table 4).

#### 2.4.8 Particle density of powder

The A/S Niro Atomiser [1,4] used was slightly modified to identify the powder's particle density (particle), as shown in Table 5.

**Table 5:** Characteristics of encapsulated powder of different formulations.

*Formulation*	*Particle density (g mL^−1^)*	*Porosity (%)*	*Oil release (%)*	
			*SGF digestion*	*SGF and SIF digestion*
AF1	0.395 ± 0.008 ^a^	62.18 ± 0.51 ^a^	18.27 ± 0.71 ^a^	46.07 ± 1.32 ^a^
AF2	0.406 ± 0.010 ^a^	64.73 ± 0.63 ^ab^	20.63 ± 0.55 ^a^	67.00 ± 2.14 ^b^
AF3	0.423 ± 0.010 ^a^	67.50 ± 0.58 ^bc^	32.33 ± 1.00 ^b^	73.73 ± 1.40 ^c^
AF4	0.435 ± 0.000 ^a^	70.18 ± 0.58 ^c^	43.63 ± 1.25 ^c^	82.10 ± 2.60 ^d^
BF1	0.493 ± 0.012 ^a^	61.91 ± 1.34 ^a^	22.00 ± 1.45 ^a^	51.83 ± 2.18 ^a^
BF2	0.443 ± 0.012 ^b^	65.02 ± 0.22 ^ab^	30.30 ± 1.11 ^b^	59.43 ± 1.88 ^b^
BF3	0.427 ± 0.006 ^bc^	65.70 ± 0.86 ^b^	53.17 ± 1.37 ^c^	68.03 ± 1.31 ^c^
BF4	0.393 ± 0.012 ^c^	70.70 ± 0.48 ^c^	67.40 ± 1.67 ^d^	80.33 ± 0.71 ^d^

Values are average of triplicate (*n* = 3) analyses ± standard deviation.

^a,b,c,d^ Letter within each column is significantly different at *p* < 0.05 using Tukey's HSD post-hoc test.

SGF - Simulated Gastric Fluid and SIF - Simulated Intestinal Fluid.

#### 2.4.9 Bulk porosity of powder

Karim et al., [1] used tapped density (tapped) and particle density (particle) to calculate the sample's bulk porosity. In this regard, the bulk porosity of powder was reflected through the particle density (particle) and the tapped density (tapped) and illustrated in Table 5.

#### 2.4.10 In vitro simulated gastric fluid (SGF) and simulated intestinal fluid (SIF) data

As shown by Patten et al., [2] and Karim et al., [1], the in-vitro digestion in this data was conducted in two stages. Firstly, the encapsulate powder dissolved in SGF with sodium chloride and pepsin with a lower pH. The next phase involved simulating intestinal digestion where the gastric digestion components were exposed to SIF (simulated intestinal fluid) (USP, 2000; [1]). The data used the USP method [3] to prepare the SGF. In this process, pepsin (0.64 g) and sodium chloride (0.4 g) were added into ultra-pure water (180 mL) before adding HCL (1.4 mL, 36% w/v). Ultra-pure water was added to produce 200 mL of solution and the pH was maintained at ∼1.2. Then, the SIF media was ready by dissolving 0.25 g of pancreatin and 1.36 g of potassium dihydrogen phosphate into ultra-pure water. The pH was adjusted using Sodium chloride (15.4 mL, 0.2 M) up to 6.8. Similarly, to the aforementioned studies, 1 M sodium hydroxide and ultra-pure water were added to obtain 200 mL of the solution.

The powder sample (5 g) and SGF (50 mL) were mixed in a 250 mL in a flask, before being incubated at 37 °C with 100 rpm using an incubator shaker for 2 h with the addition of 1 M NaOH to keep the pH at 6.8. SIF (50 ml) was replaced to the media before it was incubated again for 3 hr in the same conditions. Petroleum ether (20 mL) was added to extract the oil released and repeated 3 times throughout this time period. In each extraction process, the solution was mixed in a flash shaker for 10 mins. Prior to this process, solvent was added to the sample solution and afterwards, the solution was placed for 15 min. Then, it was mixed, a rotary evaporator was used to remove the solvent and the percentage of oil in the sample was calculated to measure the amount of oil that can be derived from the sample, as shown in Table 5.

#### 2.4.11 Peroxide value of powder

After spray drying, the samples were transferred immediately into an amber Schutt bottle for 28 days and at 4 °C. The oxidative stability was measured in 7-day intervals, and the test were performed in triplicate. The equation below was used to calculate The PV at mEq O2/kg oil:

PV = (S - B) X N X 1000)/ W (eq. 3)

Here, S represents the sample's titration (in mL), B represent the blank (in mL), N presents the sodium thiosulfate solution the normality, and W represents sample weight (in g) (Figs. 2 and 3).

## Declaration of Competing Interest

The authors declare that they have no known competing financial interests or personal relationships that could have appeared to influence the work reported in this paper.

## Appendix Supplementary materials

Image, application 1

Image, application 2

Image, application 3

Image, application 4

Image, application 5

Image, application 6

Image, application 7

Image, application 8

Image, application 9
